# A Refined Composite Multivariate Multiscale Fluctuation Dispersion Entropy and Its Application to Multivariate Signal of Rotating Machinery

**DOI:** 10.3390/e23010128

**Published:** 2021-01-19

**Authors:** Chenbo Xi, Guangyou Yang, Lang Liu, Hongyuan Jiang, Xuehai Chen

**Affiliations:** 1 Institute of Agricultural Machinery, Hubei University of Technology, Wuhan 430068, China; xichenbo@126.com (C.X.); liulang918@126.com (L.L.); jhy18307220503@126.com (H.J.); cxh@hbut.edu.cn (X.C.); 2 Hubei Engineering Research Center for Intellectualization of Agricultural Equipment, Wuhan 430068, China

**Keywords:** RCMMFDE, JMIM, rotating machinery, fault diagnosis

## Abstract

In the fault monitoring of rotating machinery, the vibration signal of the bearing and gear in a complex operating environment has poor stationarity and high noise. How to accurately and efficiently identify various fault categories is a major challenge in rotary fault diagnosis. Most of the existing methods only analyze the single channel vibration signal and do not comprehensively consider the multi-channel vibration signal. Therefore, this paper presents Refined Composite Multivariate Multiscale Fluctuation Dispersion Entropy (RCMMFDE), a method which extracts the recognition information of multi-channel signals with different scale factors, and the refined composite analysis ensures the recognition stability. The simulation results show that this method has the characteristics of low sensitivity to signal length and strong anti-noise ability. At the same time, combined with Joint Mutual Information Maximisation (JMIM) and support vector machine (SVM), RCMMFDE-JMIM-SVM fault diagnosis method has been proposed. This method uses RCMMFDE to extract the state characteristics of the multiple vibration signals of the rotary machine, and then uses the JMIM method to extract the sensitive characteristics. Finally, different states of the rotary machine are classified by SVM. The validity of the method is verified by the composite gear fault data set and bearing fault data set. The diagnostic accuracy of the method is 99.25% and 100.00%. The experimental results show that RCMMFDE-JMIM-SVM can effectively recognize multiple signals.

## 1. Introduction

Rotating machinery plays an important role in the modern industry with a complex dynamic system. During its operation, its bearings and gears may fail due to fatigue, wear and corrosion. Due to the influence of friction, load and impact, its vibration signal often appears unsteady and nonlinear characteristics [1,2,3]. Therefore, a variety of nonlinear signal analysis methods have been widely used in bearing fault diagnosis. Due to its unique advantages in feature extraction, more and more attention has been paid to entropy by researchers in ever-increasing fields, and a series of research achievements have been made [4,5,6].

Yan et al. used Approximate Entropy (APE) [7] to extract bearing fault feature information, and the results showed that this method could effectively extract fault feature, but APE had poor stability and slow calculation speed. Han used sample entropy (SE) [8,9] for bearing fault diagnosis, but this method was easily affected by abrupt signal. Azami proposed dispersion entropy (DE) [10] to quantify the regularity of the time series. This algorithm is fast in calculation and less affected by mutation signals. Rostaghi identified the status of rolling bearing and gear by DE [11], and the results showed that the status representation of rotating machinery by DE was more stable. Subsequently, Azami et al. proposed the fluctuation dispersion entropy (FDE) [12], which considered the fluctuation of time series. FDE only describes the complexity of a nonlinear time series on a single scale, resulting in the loss of a lot of important information. Therefore, Azami et al. proposed multi-scale fluctuation dispersion entropy (MFDE) [13] by extending FDE at multiple scales. To conquer the shortcomings of the coarsening method in the multi-channel signals for MFDE, On the basis of MFDE, GAN et al. introduced the coarse-grained method and proposed the composite multi-scale wave dispersion entropy (CMFDE) [14], proving that the sliding coarse-grained method based on CMFDE has better entropy stability. Zhou et al. proposed that refined complex multi-scale fluctuation dispersion entropy (RCMFDE) [15] is stronger and more stable in extracting features, and RCMDE has a smaller dependence on the length of time series. Azami et al. proposed multi-variable multi-scale dispersion entropy (MMDE) [16] in order to quantify the complexity of multivariate time series. In order to analyze data consisting of more than one-time series, Ahmed et al. proposed multi-variable multi-scale sample entropy (mvMSE) [17]. mvMSE considered both time domain and space domain, reflecting the complexity of multi-channel signals.

In order to solve the shortage of RCMFDE in multivariable time series and the poor stability of mvMSE in feature extraction, in this paper, a refined composite multivariate multiscale fluctuation dispersion entropy is proposed. This method synthesizes the information of multiple coarse-grained sequences in each channel of the multi-variable time series, and uses a refine composite method to make it less dependent on the length of the time series and more stable and reliable in feature extraction. Meanwhile, a fault diagnosis method based on Refined Composite Multivariate Multiscale Fluctuation Dispersion Entropy, Joint Mutual Information Maximisation, and Support Vector Machine (RCMMFD-JMIM-SVM) is proposed. In this method, RCMMFDE extracts the multi-variable time-series information of rotating machinery faults, uses JMIM to extract sensitive features, reduce feature dimension, and reduce the time of fault diagnosis. Finally, the SVM classifier is used to identify the fault state of rotating machinery. Two experimental results show that this method can achieve good recognition accuracy.

The rest of this paper is organized as follows. Section 2 introduces the refined complex multi-variable multi-scale fluctuation dispersion entropy method, the correlation characteristic analysis of this method, and introduces the RCMMFDE-JMIM-SVM fault diagnosis method. Section 3 introduces the diagnosis results of RCMMFDE-JMIM-SVM in different rotating machinery fault data sets and discusses relevant results. Finally, the conclusion is drawn in Section 4.

## 2. Refined Composite Multivariate Multiscale Fluctuation Dispersion Entropy

### 2.1. Multivariate Fluctuating Dispersion Entropy (MFDE)

In order to quantify the complexity of multivariate time series, based on the multivariate embedding theory, the fluctuation dispersion entropy (FDE) was extended to the multivariate fluctuation dispersion entropy (MFDE) [18]. For a multiple time series, $X={\left\{x_{k,i}\right\}}_{k=1,2,\ldots ,n}^{i=1,2,\ldots ,N}$. The detailed steps of MFDE of *X* are as follows:

(1) The multivariate time series $X={\left\{x_{k,i}\right\}}_{k=1,2,\ldots ,n}^{i=1,2,\ldots ,N}$ is mapped to $Y={\left\{y_{k,i}\right\}}_{k=1,2,\ldots ,n}^{i=1,2,\ldots ,N}$ using a normal distribution function
  (1) $$y_{k,i}=\frac{1}{\sigma_{k}\sqrt{2\pi }}\overset{x_{k,i}}{\underset{-\infty }{\int }}e^{-\frac{t-\mu_{k}}{2\sigma_{k}^{2}}}dt$$
  where μ is the expected value and $\sigma^{2}$ is the variance.
(2) The linear transformation maps *Y* to $Z={\left\{z_{k,i}\right\}}_{k=1,2,\ldots ,n}^{i=1,2,\ldots ,N}$. $0\leq z_{k,i}\leq c$
  (2) $$z_{k,i}=int\left(c\cdot y_{k,i}+0.5\right)$$
  where *c* is an integer and *int*(·) represents the rounding function. According to the multi-variable embedding theory, the embedding vector $G_{m}\left(j\right)$ is calculated by:
  $$G_{m}\left(j\right)=[z_{1,j},z_{1,j+\tau_{1}},\ldots ,z_{1,j+\left(m_{1}-1\right)\tau_{1}},\;z_{2,j},z_{2,j+\tau_{2}},\ldots ,z_{2,j+\left(m_{2}-1\right)\tau_{2}},\;\ldots ,\;z_{n,j},z_{n,j+\tau_{n}},\ldots ,z_{n,j+\left(m_{n}-1\right)\tau_{n}}]$$
  where j=1,2,…,N−(m−1)τ. The embedding dimension vector $m=\left[m_{1},m_{2},\ldots ,m_{n}\right]$, The delay coefficient vector $\tau =\left[\tau_{1},\tau_{2},\ldots ,\tau_{n}\right]$. Convert $G_{m}\left(j\right)$ to $F_{m}\left(j\right)$,
  $$F_{m}\left(j\right)=\{z_{1,j+\tau_{1}}-z_{1,j}+c,\ldots ,z_{1,j+\left(m_{1}-1\right)\tau_{1}}-z_{1,j+\left(m_{1}-2\right)\tau_{1}}+c,\;z_{2,j+\tau_{2}}-z_{2,j}+c,\ldots ,z_{2,j+\left(m_{2}-1\right)\tau_{2}}-z_{2,j+\left(m_{2}-2\right)\tau_{2}}+c,\;\ldots ,\;z_{n,j+\tau_{n}}-z_{n,j}+c,\ldots ,z_{n,j+\left(m_{n}-1\right)\tau_{n}}-z_{n,j+\left(m_{n}-2\right)\tau_{n}}+c,\}$$

For every $G_{m}\left(j\right)$, all combinations of *m*−1 elements in $G_{m}\left(j\right)$ are called $\phi_{q,l}\left(j\right)$, where $q\in \left[1,C_{m-1}^{\left(m-1\right)n}\right]$, l∈[1,m−1]. Map each $\phi_{q,l}\left(j\right)$ to a scatter pattern $\pi_{v_{0}v_{1}\ldots v_{m-2}}\left(v=1,2,\ldots ,c\right)$. In this mode, $\phi_{q,1}\left(j\right)=v_{0}$, $\phi_{q,1}\left(j\right)=v_{1}$,…,$\;\phi_{q,l}\left(j\right)=v_{m-2}$. Since $\pi_{v_{0}v_{1}\ldots v_{m-2}}$ consists of *m*−1 digits and each m−1 has class c, there are c(m−1) dispersion patterns. the total number of combinations per $G_{m}\left(j\right)$ is $C_{m-1}^{\left(m-1\right)n}$. Therefore, there are $\left[n-\left(m-1\right)d\right]C_{m-1}^{\left(m-1\right)n}$ dispersion patterns for time series with n variables. Calculate the probability of each distribution mode $\pi_{v_{0}v_{1}\ldots v_{m-2}}$ by:

(3) $$P\left(\pi_{v_{0}v_{1}\ldots v_{m-2}}\right)=\frac{Num\left(\pi_{v_{0}v_{1}\ldots v_{m-2}}\right)}{\left(N-\left(m-1\right)d\right)C_{m-1}^{\left(m-1\right)n}}$$

(3) According to the definition of Shannon entropy, the multivariate fluctuation dispersion entropy of the original signal X is:
  (4) $$MFDE\left(X,m,c,\tau \right)=-\overset{{\left(2c-1\right)}^{m-1}}{\underset{\pi =1}{\sum }}P\left(\pi_{v_{0}v_{1}\ldots v_{m-2}}\right)InP\left(\pi_{v_{0}v_{1}\ldots v_{m-2}}\right)$$

### 2.2. Multivariate Multiscale Fluctuation Dispersion Entropy (MMFDE)

The steps of the MMFDE are first to establish the coarse-grained time series of the original multivariate signal and then calculate the MFDE for each coarse-grained multivariate time series. For a multiple time series, $U={\left\{\mu_{k,b}\right\}}_{k=1,2,\ldots ,N}^{b=1,2,\ldots ,L}$. The detailed steps of MFDE of U are as follows:

(1) For a multivariable time series, $U={\left\{\mu_{k,b}\right\}}_{k=1,2,\ldots ,N}^{b=1,2,\ldots ,L}$ with length L and number of signal channels N. Then, the coarse granulation time series of scale factor τ is:
  (5) $$x_{k,i}^{\tau }=\frac{1}{\tau }\overset{i\tau }{\underset{b=\left(i-1\right)\tau +1}{\sum }}\mu_{k,b},\;1\leq i\leq \frac{L}{\tau }$$
(2) MMFDE was obtained by calculating the MFDE of each coarse-grained multivariate time series $\left\{x_{k,i}^{\tau }\right\}$ under the same parameters. By extending the single-scale MFDE to the multi-scale, more information is obtained from the multi-scale coarse-grained time series of different scales to obtain the multi-scale MMFDE. However, in the coarse-grained multivariate time series whose MMFDE scale factor is τ, only the coarse-grained multivariate time series starting with $\mu_{k,1}$ is considered, and the remaining τ−1 multivariable time series is missing. The relationship between the coarse-grained time series has not been taken into consideration, resulting in the lack of statistical information.

### 2.3. Refined Composite Multivariate Multiscale Fluctuation Dispersion Entropy (RCMMFDE)

(1) For a multivariable time series $U={\left\{\mu_{k,b}\right\}}_{k=1,2,\ldots ,N}^{b=1,2,\ldots ,L}$ with length *L* and number of signal channels *N*, the a coarse-grained time series for the given scale factor τ is $X_{a}^{\tau }=\left\{x_{k,i,1}^{\tau },x_{k,i,2}^{\tau },\ldots ,\right\}$, where $x_{k,i,a}^{\tau }=\frac{1}{\tau }\overset{a+i\tau -1}{\underset{b=a+\left(i-1\right)\tau }{\sum }}\mu_{k,b}$, where $1\leq i\leq \frac{L}{\tau }$, 1≤a≤τ.
(2) The RCMMFDE of the original multivariable time series $U={\left\{\mu_{k,b}\right\}}_{k=1,2,\ldots ,N}^{b=1,2,\ldots ,L}$ is:
  (6) $$RCMMFDE\left(U,m,c,d,\tau \right)=-\overset{{\left(2c-1\right)}^{m-1}}{\underset{\pi }{\sum }}\bar{p}\left(\pi_{v_{0}v_{1}\ldots v_{m-2}}\right)In\bar{p}\left(\pi_{v_{0}v_{1}\ldots v_{m-2}}\right)$$
  where *m* is the embedding dimension, *c* is the number of categories, *d* denotes the delay coefficient, τ represent the scale factor. $\bar{p}\left(\pi_{v_{0}v_{1}\ldots v_{m-2}}\right)=\frac{1}{\tau }\overset{\tau }{\underset{1}{\sum }}p_{a}^{\tau }$ is the average probability of the dispersion mode $\pi_{v_{0}v_{1}\ldots v_{m-2}}$ of coarse-grained sequence $X_{a}^{\tau }$. $p_{a}^{\tau }$ is the frequency of scattering mode $\pi_{v_{0}v_{1}\ldots v_{m-2}}$ in the A multivariate coarse-grained time series $X_{a}^{\tau }$.

### 2.4. RCMMFDE Feature Analysis

In this section, five sets of simulation signals are used to verify the relevant characteristics of RCMMFDE. Firstly, white noise and 1/*f* noise are used for analysis to study RCMMFDE’s ability to measure the complexity of multi-variable time series and its stability under different time series lengths. Finally, the synthetic signal is used to verify the anti-noise performance of RCMMFDE.

#### 2.4.1. Mixed Analysis of White Noise and 1/f Noise

**Figure 1:**
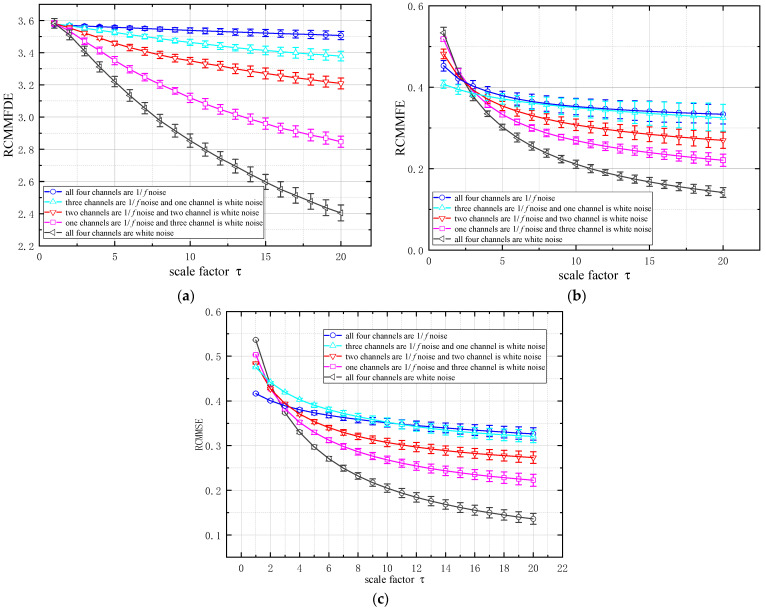
RCMMFDE, RCMMFE and RCMMSE values of different methods under different noise combinations: (**a**) RCMMFDE; (**b**) RCMMFE; (**c**) RCMMSE.

In order to illustrate the corresponding characteristics of multivariable time series, RCMMFDE is applied to the generated four-channel time series: (1) all four channels are 1/*f* noise; (2) three channels are 1/*f* noise and one channel is white noise; (3) two channels are 1/*f* noise and two channel is white noise; (4) one channels are 1/*f* noise and three channel is white noise; (5) all four channels are white noise. The parameter values used to calculate RCMMFDE in this section are set as: embedding dimension *m* = 3, category *c* = 5, delay coefficient *d* = 1, scale factor τ = 2. For the convenience of comparison, the calculated results of the 50 samples were averaged. Figure 1 shows the mean standard deviation curves of RCMMFDE, Refined Composite Multivariate Multiscale Fluctuation Entropy(RCMMFE) [19] and Refined Composite Multivariate Multiscale Sample Entropy(RCMMSE) [20]. As shown in Figure 1, the overall trend of changes in RCMMFDE, RCMMFE and RCMMSE is roughly similar, which all decrease with the increase of white noise channel and the increase of scale factor. This is because the time series consisting of 4-channel 1/*f* noise is rather complex. The complexity of time series decreases with the decrease of 1/*f* channel, and the complexity of time series composed of four channel white noise is the lowest. RCMMFE and RCMMSE overlap widely in the first category (all four channels are 1/*f* noise) and the second category (three channels are 1/*f* noise and one channel is white noise), and the separation is not obvious. but, RCMMFDE has a clear distinction between category 1 and category 2 with a lower standard deviation. Which proves that RCMMFDE has better resolution ability and stability and more suitable for the multi-channel signal feature extraction.

#### 2.4.2. Analysis of Anti-Noise Performance

**Figure 2:**
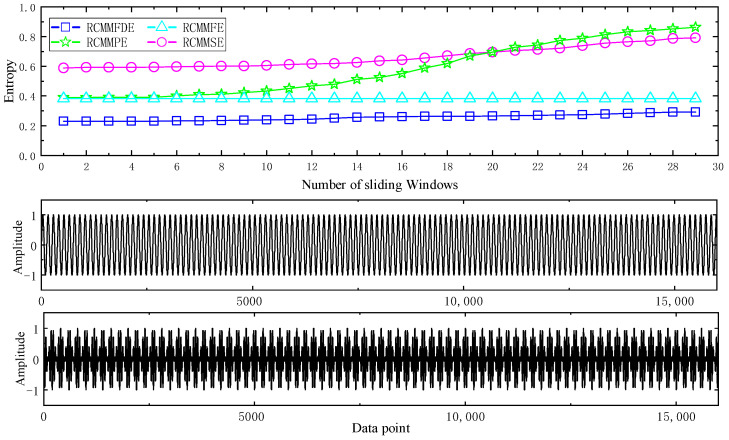
RCMMFDE of containing periodic signals of varying noise.

In order to study the anti-noise performance of different entropy algorithms, a two-channel composite signal with noise power variation is adopted. Each channel has a sampling frequency of 512Hz and a length of 32s. To make a fair comparison, the embedded dimensions in RCMMFDE, RCMMFE, RCMMSE and RCMMPE are set to equal *m* = 3, and *r* = 0.15 in RCMMFE and RCMMSE set the fuzzy power of RCMMFE to *n* = 2. The sensitivity of RCMMFDE to noise was studied by using the dual-channel periodic signal with noise power variation. This periodic signal had no noise in the first 4 s, and white noise was added after 4 s, and the noise power was increased every 1 s. In order to reduce the influence of entropy on the visualization of entropy change, the entropy values of different methods are scaled to the range of 0–1, as shown in Figure 2. As shown in Figure 2, the values of RCMMSE and RCMMPE increase rapidly with the increase of noise power, but RCMMPE increases faster than RCMMSE. The curve of RCMMFDE grows slowly, but RCMMFE performs better in variable noise synthesis signals. The results show that RCMMFE and RCMMFDE have better anti-noise capability.

#### 2.4.3. RCMMFDE Data Length Sensitivity Analysis

**Figure 3:**
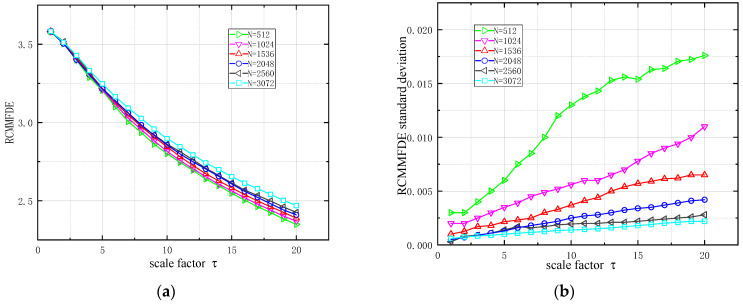
Comparison of white noise with different data lengths: (**a**) Mean value of RCMMFDE; (**b**) Standard deviation of RCMMFDE.

In order to compare the stability of RCMMFDE under different data lengths, four-way white noise samples with data lengths of 512, 1024, 1536, 2048, 2560 and 3072 were used, and the number of samples of each data length was 100. RCMMFDE was calculated 20 times for each data length, and its mean value and standard deviation are shown in Figure 3. As shown in Figure 3a, as the data length increases, the value of RCMMFDE is relatively close. However, when the scale factor is greater than 5, the RCMMFDE with lower data length is slightly smaller than the RCMMFDE with higher data length, and this gap will increase with the increase of the scale factor. This indicates that RCMMFDE is less sensitive to data length and can obtain reliable measurements of samples of different data lengths. As shown in Figure 3b, the standard deviation of RCMMFDE increases with the increase of scale factor and the decrease of data length. In particular, when the data length is 500, the standard deviation increases significantly. When the data length is 3000, the standard deviation of RCMMFDE is the smallest, which indicates that the longer the data length is, the more stable RCMMFDE is. However, the data length is positively correlated with the computation time. Given the tradeoff between RCMMFDE stability and computation time, the data sample length is set to 2048.

### 2.5. Fault Diagnosis Based on RCMMFDE

#### 2.5.1. JMIM Feature Selection

Joint Mutual Information Maximisation (JMIM) [21] is an effective feature selection algorithm. Considering the overall stability of Joint Mutual Information, JMIM is able to select a subset of features with the same or even better effect from the original feature set [22]. Compared with other algorithms, such as mRMR, JMI and IG, the feature subset selected by JMIM algorithm has better classification performance [23]. Therefore, the JMIM algorithm is used as the sensitive sub-feature selection method in this paper.

#### 2.5.2. RCMMFDE-JMIM-SVM Fault Diagnosis Algorithm

**Figure 4:**
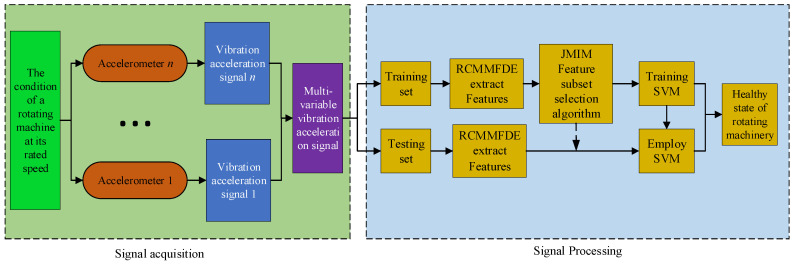
RCMMFDE-JMIM-SVM fault diagnosis flow chart.

In many fault diagnosis fields, Support Vector Machine (SVM) has been proved to be robust nonlinear multiple classifiers [24,25]. Combined with the advantages of RCMMFDE, JMIM, and SVM, this paper proposes a fault diagnosis method of rotating machinery based on RCMMFDE-JMIM-SVM. The flow chart is shown in Figure 4.

Step 1. Signal acquisition: Through acceleration sensors installed at different positions of the rotating machinery to be monitored, multi-channel vibration signals of the rotating machinery under different speeds are collected. The data samples collected under different running speeds can form a fault sample data set.
Step 2. Feature extraction: RCMMFDE algorithm is used to extract features from fault sample data to form feature data sets, which are divided into training data sets and test data sets.
Step 3. Feature selection: JMIM algorithm is used to select the most sensitive sub-features of the feature.
Step 4. SVM model training: SVM classifier is trained using sensitive features. The SVM classifier includes M-1 dichotomous SVM, where M is the type of fault sample.
Step 5. Fault diagnosis: Input the samples of the test data set into the RCMMFDE-JMIM-SVM classifier to identify the fault types of different bearing samples.

## 3. Experimental Verification and Analysis

In this paper, a gearbox fault data set and a rolling bearing fault data set are respectively used to verify the effectiveness of the method. Collect the multivariable time series of gearbox and rolling bearing at constant speed. RCMMFE, RCMMSE, RCMMPE and RCMMFDE were compared, and the good performance of the method was verified by the identification accuracy and CPU time.

### 3.1. Validation of Gearbox Fault Data Set

#### 3.1.1. Data Set Description

**Figure 5:**
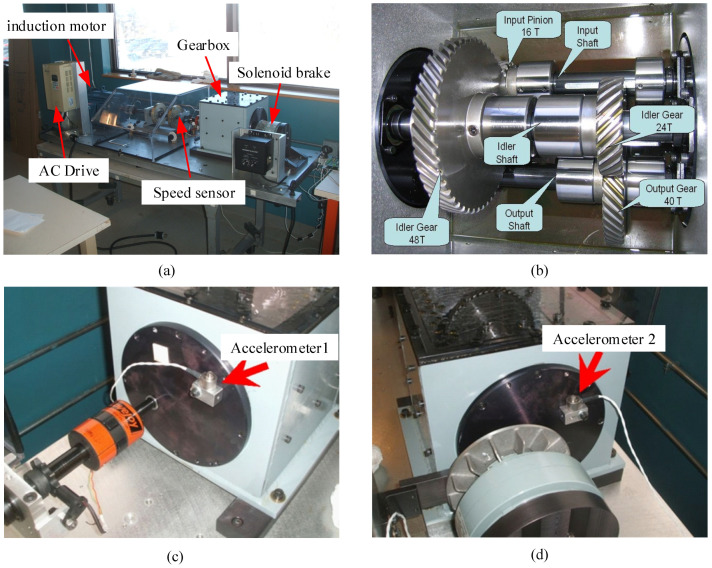
Gearbox fault test platform: (**a**) gearbox test bench; (**b**) internal structure of gearbox; (**c**) installation position of accelerometer 1; (**d**) installation position of accelerometer 2.

**Figure 6:**
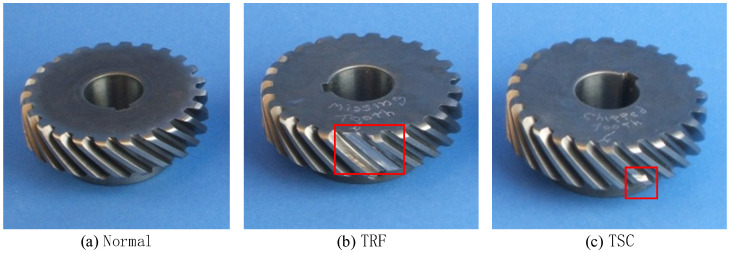
Gear fault type: (**a**) Normal; (**b**) TRF; (**c**) TSC.

**Figure 7:**
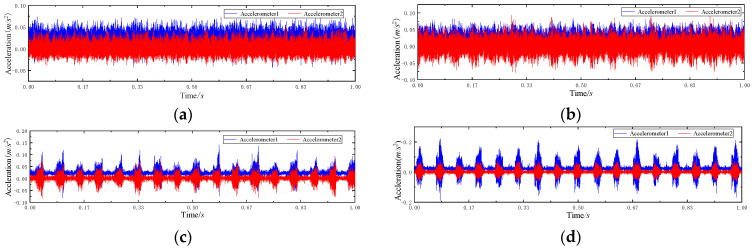

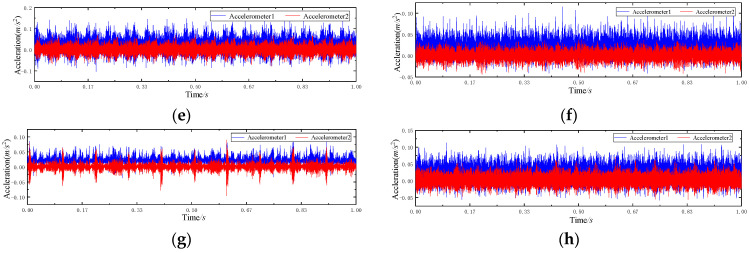
Time domain waveform of vibration signal: (**a**) G1; (**b**) G2; (**c**) G3; (**d**) G4; (**e**) G5; (**f**) G6; (**g**) G7; (**h**) G8.

This data set is the 2009 PHM Challenge data set (in Supplementary Materials), which is the gearbox composite failure test data obtained on the gearbox test bench. The test platform is shown in Figure 5a, which mainly includes induction motor, gearbox, speed measuring device, solenoid brake, AC drive, experimental bench and data acquisition system. The gearbox includes input shaft, intermediate shaft and output shaft. The internal structure diagram is shown in Figure 5b. Two accelerometers are placed at the input end and output end of the gearbox respectively to collect vibration signals of the gear box, as shown in Figure 5c,d. This paper takes the meshing fault of helical gear as the research object, Based on Tooth Surface Crack (TSC), Tooth Root Fracture (TRF), Bearing Outer Fault (BOF), Bearing Inner Outer Fault (BIF), Bearing Balling Fault, As for fault types such as BBF), Gear Imbalance (GI) and Input Shaft Imbalance (ISI), a result of eight helical gears meshing composite fault states is simulated. Information about the composite fault state is illustrated in Table 1, and Gear of each fault is illustrated in Figure 6. The sampling frequency is 66.67 KHz, the rated speed of input shaft is 3000 r/min, and the sampling time is 4 s gear box vibration signal under each compound fault state. The signal is divided into several samples, each with a length of 2048. The labels and sample Numbers are shown in Table 2. The time-domain waveform of the gear in different states is shown in Figure 7. Because there are many fault states, it is difficult to distinguish the specific bearing fault from the time domain waveform.

#### 3.1.2. RCMMFDE-JMIM-SVM Sensitive Feature Number Analysis

**Figure 8:**
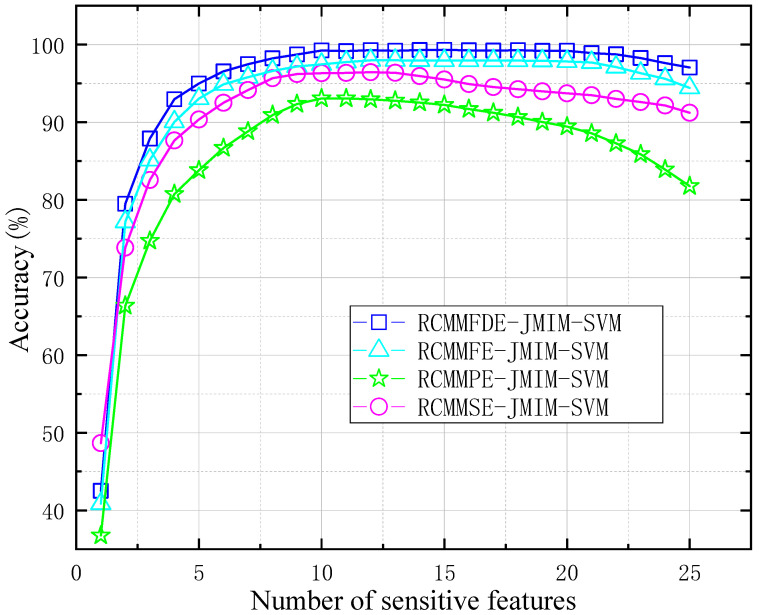
Average diagnostic accuracy under different number of sensitive features.

In each data sample of the above data set, 200 samples were randomly selected as test objects, of which 150 were training samples and 50 were test samples. The sensitive feature dimension p extracted by JMIM was set as 25. In order to verify the performance of the RCMMFDE-JMIM-SVM method, RCMMFDE, RCMMFE, RCMMSE, and RCMMPE were used to extract the features of vibration signals, and the optimal features were extracted through the JMIM algorithm. Finally, the optimal features were input into the SVM classifier to identify the fault type of the test sample. Each method was run 20 times respectively. The specific parameters of RCMMFDE can be known from Azami research that the number of categories *c* = 6, the delay coefficient *d* = 1, the embedding dimension *m* = 2, and the maximum scale factor τ = 25 [26]. The average diagnostic accuracy of different methods is shown in Figure 8. As can be seen from Figure 8, the recognition accuracy of RCMMFDE-JMIM-SVM method is better than other methods, because RCMMFDE takes into account the amplitude changes between two adjacent elements at different scales of each channel in the multi-channel time series. Secondly, too large or too small number of sensitive features will reduce the accuracy of fault recognition. This is because too small number of feature sensitivities will contain less fault feature information. On the contrary, too large number of sensitive features will lead to redundancy of fault feature information and reduce fault identification accuracy. Secondly, too large or too small several sensitive features will reduce the accuracy of fault recognition. When the number of sensitive features is less than 21, the accuracy of the RCMMFDE-JMIM-SVM method will increase with the increase of the number of sensitive features and eventually tend to be stable. When the number of sensitive features is greater than 21, the accuracy of the model will decrease with the increase in the number of sensitive features. Therefore, this paper sets the number of sensitive features at 20.

#### 3.1.3. RCMMFDE-JMIM-SVM Performance Analysis at Constant Speed

To verify the effectiveness of the proposed method under the condition of constant rotation speed, RCMMFDE-JMIM-SVM, RCMMFE-JMIM-SVM, RCMMSE-JMIM-SVM, and RCMMPE-JMIM-SVM methods were used to analyze the gear fault data set. The main parameters of these methods include embedding dimension *m*, number of categories *c*, delay coefficient *d*, fuzzy power *f*, scale factor τ, threshold *r* and characteristic sensitive number *p*. For the sake of fair comparison, the scale factors of these methods are consistent, and the specific parameter settings are shown in Table 3.

**Figure 9:**
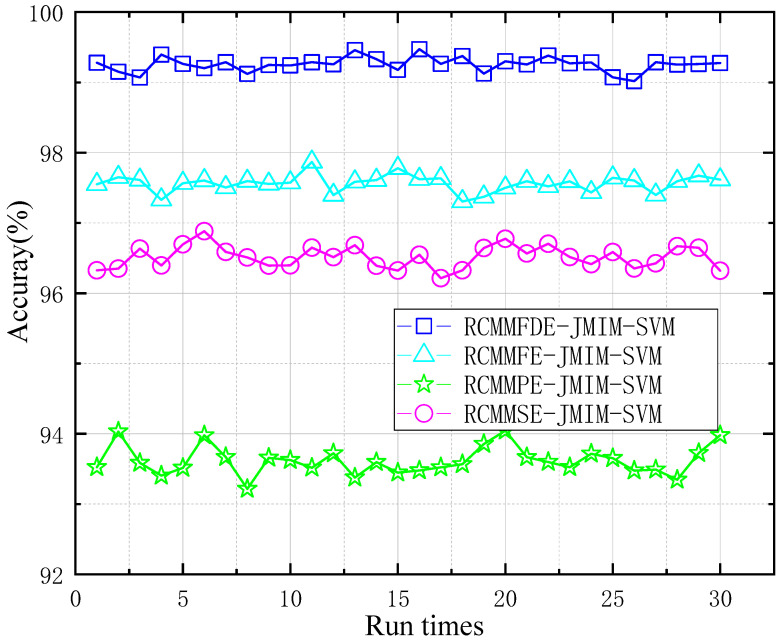
Diagnostic accuracy of different diagnostic methods.

Figure 9 and Table 4 show the identification accuracy of four different fault diagnosis methods within 30 running times. The computers used in this paper are configured as Windows 10 operating system, Intel Core i5-9400f CPU and 16 G RAM. As is shown in Figure 9, The average accuracy of RCMMFDE-JMIM-SVM is 98.757%, and the maximum classification accuracy is 99.252%. This shows that the method is effective for the diagnosis of gear fault data set. The classification accuracy of RCMMFE-JMIM-SVM is between 97.867% and 97.305%. Compared with the method presented in this paper, the diagnostic accuracy of RCMMFE-JMIM-SVM is slightly lower. The classification accuracy of RCMMPE-JMIM-SVM method was the lowest, between 93.217% and 94.045%. Furthermore, the processing time of RCMMFDE-JMIM-SVM for a single sample was 10.32s, 0.53s slower than that of RCMMSE-SVM, which was nearly 90 times slower than that of RCMMFDE-JMIM-SVM. The reason why RCMMFDE-JMIM-SVM achieves excellent performance is that the fluctuation between adjacent elements of each channel of the multivariate time series is considered, and the features can be extracted from the multivariate time series between different signals, and the influence of noise and fluctuation can be resisted.

**Figure 10:**
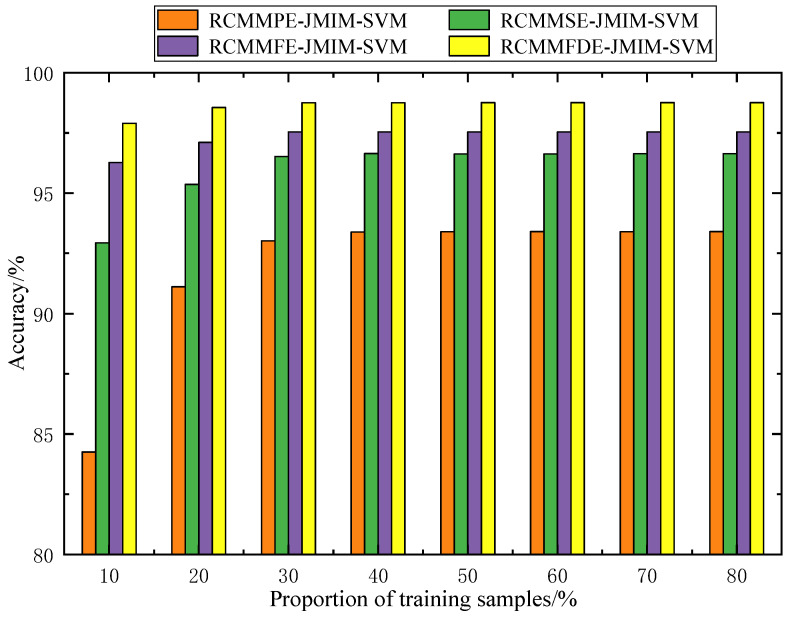
Recognition accuracy of different training sample proportions.

To analyze the relationship between the recognition accuracy and the number of training samples, we randomly selected training samples from the total samples under different proportions (10%, 20%, 30%, 40%, 50%, 60%, 70%, 80%) and calculated the average recognition accuracy of different diagnostic methods in the running time of 50 times respectively. Figure 10 shows the average classification accuracy under different proportions of training samples. As shown in Figure 10, the accuracy of different classification methods increases with the proportion of training samples. Although the proportions of training samples are different, RCMMFDE-JMIM-SVM can achieve the highest diagnostic accuracy. Even if 10% of the samples are used for training, the diagnostic accuracy can reach 97.896%, and when the proportion of training samples increases to 30%, the accuracy reaches 98.961%. It can be found that if sufficient training data samples are available, all the four methods can achieve high average diagnostic accuracy. However, too many training samples will lead to a significant increase in training time. Therefore, to balance the training time and accuracy, the proportion of training samples was set at 40%.

#### 3.1.4. RCMMFDE-JMIM-SVM Performance Verification

To better present the performance of RCMMFDE-JMIM-SVM, this paper also uses the common feature of vibration signal and the SVM (CFVS-SVM) to verify performance of proposed method. Common features we take in this paper are time-domain features, frequency-domain features, and time-frequency features. We take 18 features including mean value, standard deviation, peak-to-peak value, median, interquartile range and etc. as the time domain features. The frequency-domain features are median mean, interquartile range, percentile deviation, and Fourier transform features. The time-frequency domain features are the six energy features and their normalized energy features obtained by the five-stage wavelet transform.

Table 5 shows the results of four entropy fault diagnosis methods and CFVS-SVM diagnosis methods trained with 40% training samples. The classification accuracy of Table 5 is the average value of 20 times’ result. The Table 5 shows that in G2, G4 and G5 and G8 condition, CFVS-SVM and RCMMPE-JMIM-SVM accuracy have plunged, the accuracy of the RCMMSE-JMIM-SVM and RCMMFE-JMIM-SVM have appeared in the different amplitude of falling, but in this paper, fault identification accuracy of the presented method still remain more than 99%, fully shows in different fault conditions, the proposed method of feature extraction is better than the other three methods.

**Figure 11:**
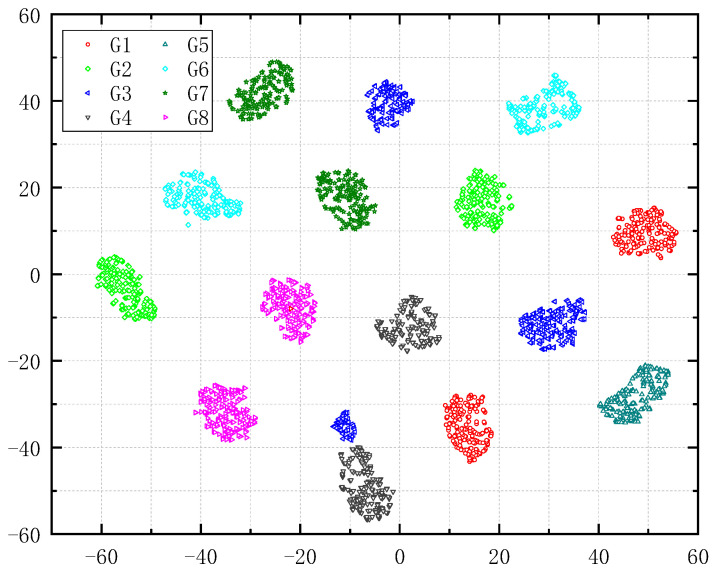
Visualized by T-SNE features.

In order to demonstrate the effectiveness of RCMMFDE-JMIM feature extraction, Figure 11 shows the feature distribution visualization diagram of all samples extracted by RCMMFDE-JMIM algorithm by T-SNE method [27]. It can be seen from Figure 11 that each fault sample has a high degree of intra-class clustering and inter-class separation, but the distance between classes is small in some states of G3 and G4, which leads to the relatively low accuracy of this method in G3 state, but it does not affect the performance of RCMMFDE-JMIM-SVM method. In order to demonstrate the effectiveness of RCMMFDE-JMIM feature extraction, Figure 11 shows the feature distribution visualization diagram of all samples extracted by the RCMMFDE-JMIM algorithm by the T-SNE method [28]. It can be seen from Figure 11 that each fault sample has a high degree of intra-class clustering and inter-class separation, but the distance between classes is small in some states of G3 and G4, which leads to the relatively low accuracy of this method in the G3 state, but it does not affect the performance of RCMMFDE-JMIM-SVM method.

### 3.2. Verification of Rolling Bearing Fault Data

#### 3.2.1. Description of Rolling Bearing Fault Data Set

**Figure 12:**
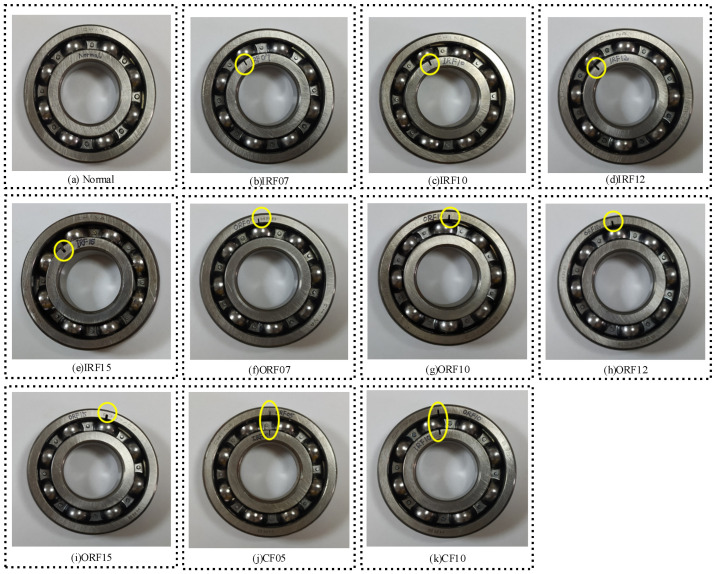
Different types of faulty bearings.

**Figure 13:**
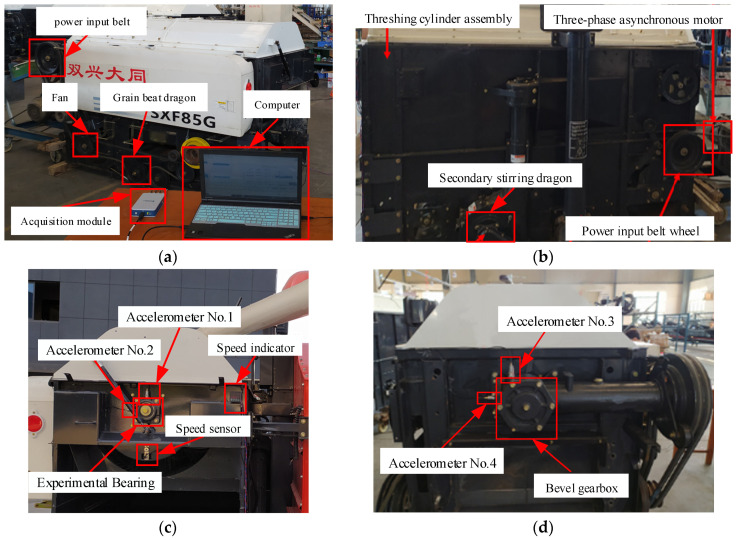
Schematic diagram of experimental platform and sensor installation: (**a**) threshing cylinder front view; (**b**) threshing cylinder back view; (**c**) sensor mounting position 1; (**d**) sensor mounting position 2.

**Figure 14:**
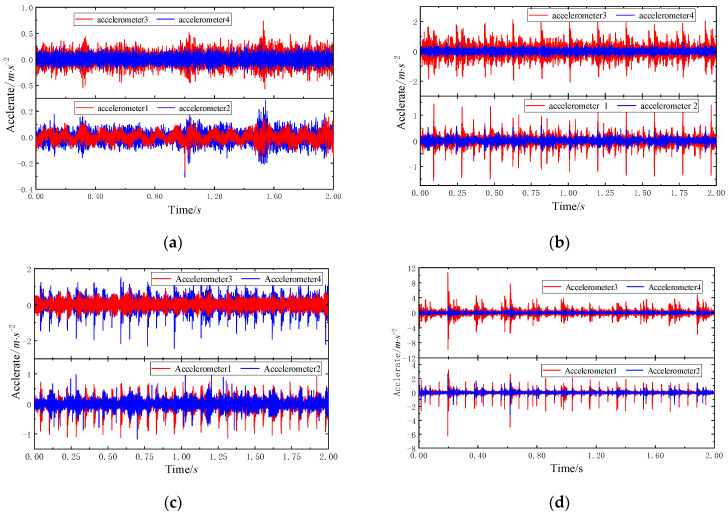
Time domain waveform of four different states: (**a**) normal; (**b**) IRF15; (**c**) ORF15; (**d**) CD10.

The test platform is the threshing cylinder part of the combine harvester, which is mainly composed of the motor driving part, threshing assembly, and data acquisition system. The test bearing is the 6307 deep-groove ball bearing at the back end of the threshing roller. The outer ring of the bearing is fixed on the bearing pedestal, and the inner ring rotates with the threshing roller shaft. Other bearings of the system are normal bearings. In the test, three common fault types of 6307 deep-groove ball bearings were considered, including Inner Ring Fault (IRF), Outer Ring Fault (ORF), and Composite Fault (CF) occurring simultaneously in the inner ring. The detailed parameters of each fault are shown in Table 6. Bearing faults of different types and sizes are simulated by EDM pitting technique. Figure 12 shown rolling bearings with combined faults of inner ring, outer ring and inner ring, respectively. The acceleration sensor is fixed at the measuring point through the magnetic base. The voltage sensitivity of the 1# to 4# acceleration sensors is 101.6 mV/g, 99.1 mV/g, 101.2 mV/g, and 101.3 mV/g, respectively. The installation locations of acceleration sensors and speed sensors are shown in Figure 13. The threshing cylinder rotation speed was set at 80 r/min, 160 r/min, 240 r/min, and 320 r/min to collect the bearing vibration signal through the data acquisition system, the sampling frequency is 5120 Hz, each group of sampling time 50 s, The sampling number of each sample is 2048, each bearing status get 500 samples, a total of 5500 samples. The time domain waveform of the original four-channel signals of different fault types is shown in Figure 14.

#### 3.2.2. Rolling Bearing Fault Data Analysis

The 100 samples of each bearing state are taken as training data, and the other 25 samples are taken as test data, thus forming the rolling bearing fault data set. In the rolling bearing fault data analysis, the RCMMFDE-JMIM-SVM method parameters determined in 3.1 are used. The comparison results of RCMMFDE-JMIM-SVM, RCMMFE-JMIM-SVM, RCMMSE-JMIM-SVM, RCMMPE SVM and CFVS-SVM are shown in Table 7. As can be seen from Table 7, the accuracy of all methods on the rolling bearing data set is significantly higher than that on the gear data set. The accuracy of RCMMFDE-JMIM-SVM and RCMMFE-JMIM-SVM reaches 100%, and the accuracy of RCMMSE-JMIM-SVM RCMMPE-JMIM-SVM and CFVS-SVM is also greater than 96%. The reasons for this phenomenon may be the relatively ideal test environment and the good performance of the data acquisition system, which makes the characteristics of the signal data more easily to identify. It also shows that RCMMFDE-JMIM-SVM has good performance.

**Figure 15:**
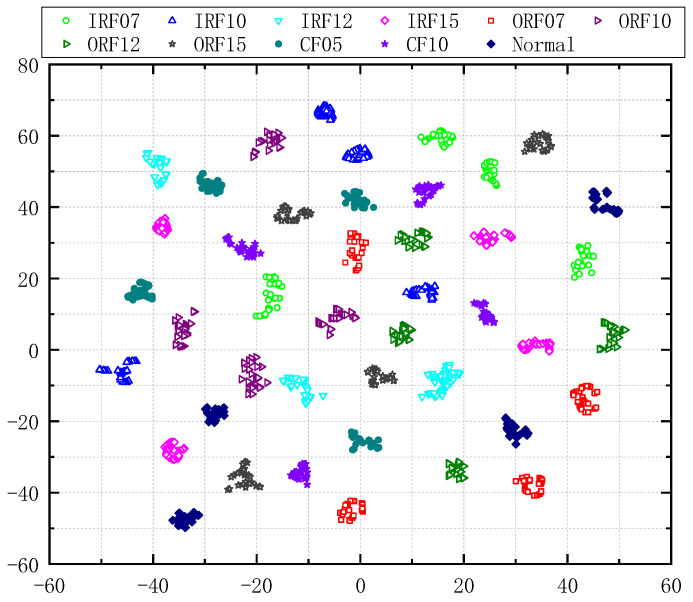
Visualized features by T-SNE.

To demonstrate the effectiveness of RCMMFDE-JMIM-SVM in the bearing fault data set, Figure 15 shows the visualization of the feature distribution of all samples by t-SNE method. It can be seen from Figure 15 that the separation between classes of each fault feature is high and the separation is also high at different speeds of different faults.

## 4. Conclusions

In this paper, a new complexity assessment method of multivariable nonlinear time series based on RCMMFDE is proposed. Compared with RCMMFE, RCMMSE, and RCMMPE, RCMMFDE has a better stability. This method takes into account the information of different scale sequences of each channel in the original multicomponent signal and obtains more stable and reliable characteristics by using the fine composite analysis technique. Use the JMIM method to select the more sensitive feature. In the classification step, the SVM classifier is trained to classify sensitive feature samples. This method can effectively process multivariate time series data.

The simulation signals with different combinations of WGN and 1/*f* show that RCMMFDE has better resolution and stability. The research on the two-channel stimulation signal of noise power variation shows that RCMMFDE has excellent anti-noise performance. Meanwhile, the use of four-channel WGN signal indicates that RCMMFDE is not sensitive to the length of time series signal. Taking gear fault data set and bearing fault data set as examples, the effectiveness of RCMMFDE-JMIM-SVM method is verified. The results show that this method has the advantages of high classification accuracy and short calculation time.

The research in this paper shows that the RCMMFDE-JMIM-SVM method is suitable for the diagnosis of gear and bearing faults, and the effectiveness of this method on other data sets can be further studied.

## Figures and Tables

**Table 1:** Compound fault status information of helical gear meshing.

Abbreviation		G1	G2	G3	G4	G5	G6	G7	G8
Shaft	Input	Normal	Normal	Normal	Normal	Normal	ISI	Normal	ISI
	Output	Normal	Normal	Normal	Normal	Normal	Normal	Keyway wear	Normal
Bearing	Input shaft—Input end	Normal	Normal	Normal	BBF	BIF	BIF	BIF	Normal
	Intermediate shaft— Input shaft	Normal	Normal	Normal	Normal	BBF	BBF	Normal	BBF
	Output shaft—Input end	Normal	Normal	Normal	Normal	BOF	BOF	Normal	BOF
	Input shaft—Output end	Normal	Normal	Normal	Normal	Normal	Normal	Normal	Normal
	Intermediate shaft— Output end	Normal	Normal	Normal	Normal	Normal	Normal	Normal	Normal
	Output shaft—Output end	Normal	Normal	Normal	Normal	Normal	Normal	Normal	Normal
Gear	32T	Normal	TSC	Normal	Normal	TSC	Normal	Normal	Normal
	96T	Normal	Normal	Normal	Normal	Normal	Normal	Normal	Normal
	48T	Normal	GI	GI	GI	GI	Normal	Normal	Normal
	80T	Normal	Normal	Normal	TRF	TRF	TRF	Normal	Normal

**Table 2:** Gearbox fault data set label.

No	State	Training Samples	Testing Samples	Class Label
1	G1	200	100	1,0,0,0,0,0,0,0
2	G2	200	100	0,1,0,0,0,0,0,0
3	G3	200	100	0,0,1,0,0,0,0,0
4	G4	200	100	0,0,0,1,0,0,0,0
5	G5	200	100	0,0,0,0,1,0,0,0
6	G6	200	100	0,0,0,0,0,1,0,0
7	G7	200	100	0,0,0,0,0,0,1,0
8	G8	200	100	0,0,0,0,0,0,0,1

**Table 3:** Parameter setting table.

Method	Parameter					
RCMMFE-JMIM-SVM	p = 17	*m* = 2	*r* = 0.15	*d* = 1	τ = 25	*f = 2*
RCMMSE-JMIM-SVM	p = 12	*m* = 2	*r* = 0.15	*d* = 1	τ= 25	
RCMMPE-JMIM-SVM	p = 17	*m* = 2	τ = 17			
RCMMFDE-JMIM-SVM	p = 20	*m* = 2	*c =* 6	*d* = 1	τ = 25	

**Table 4:** Diagnostic accuracy of different diagnostic methods.

Method	Max Accuracy	Min Accuracy	Average Accuracy	Run Time
RCMMFDE-JMIM-SVM	99.252	98.518	98.757	1.67
RCMMSE-JMIM-SVM	96.882	96.215	96.648	1.14
RCMMPE-JMIM-SVM	94.045	93.217	93.554	148.79
RCMMFE-JMIM-SVM	97.867	97.305	97.694	8.28

**Table 5:** Different methods each fault state accuracy rate.

State	Testing Samples	Accuracy/%				
		RCMMFDE-JMIM-SVM	RCMMFE-JMIM-SVM	RCMMSE-JMIM-SVM	RCMMPE-JMIM-SVM	CFVS-SVM
G1	180	99.425	98.627	98.129	96.951	97.273
G2	180	99.354	97.056	97.281	91.488	94.445
G3	180	97.812	95.627	98.835	97.538	97.758
G4	180	99.489	97.566	95.756	92.157	95.561
G5	180	99.105	97.634	96.018	92.266	93.891
G6	180	99.281	98.461	96.485	94.137	96.659
G7	180	99.577	97.343	97.528	94.513	96.562
G8	180	98.907	96.932	94.414	93.064	94.827
Total	900	99.173	97.782	96.619	93.751	94.658

**Table 6:** Table of parameters of different fault state of bearing.

Bearing State	Abbreviation	Fault Width (mm)	Fault Depth (mm)	Threshing Cylinder Speed (r/min)
Normal	Normal	0	0	80/160/240/320
Outer Ring Fault 1	IRF07	0.7	3.7	80/160/240/320
Outer Ring Fault 2	IRF10	1.0	3.7	80/160/240/320
Outer Ring Fault 3	IRF12	1.2	3.7	80/160/240/320
Outer Ring Fault 4	IRF15	1.5	3.7	80/160/240/320
Outer Ring Fault 1	ORF07	0.7	3.2	80/160/240/320
Outer Ring Fault 2	ORF10	1.0	3.2	80/160/240/320
Outer Ring Fault 3	ORF12	1.2	3.2	80/160/240/320
Outer Ring Fault4	ORF15	1.5	3.2	80/160/240/320
Composite Fault 1	CF05	0.5/0.5	3.2/3.7	80/160/240/320
Composite Fault 2	CF10	1.0/1.0	3.2/3.7	80/160/240/320

**Table 7:** The results of different diagnostic methods were compared.

Bearing State	Testing Samples	RCMMFDE-JMIM-SVM		RCMMFE-JMIM-SVM		RCMMSE-JMIM-SVM		RCMMPE-JMIM-SVM		CFVS-SVM	
		MCR	Acc/%	MCR	Acc/%	MCR	Acc/%	MCR	Acc/%	MCR	Acc/%
Normal	25	0	100.00	0	100.00	0	100.00	0	100.00	CF05	96.00
IRF07	25	0	100.00	0	100.00	IRF10	96.00	IRF12	88.00	IRF10	92.00
IRF10	25	0	100.00	0	100.00	0	100.00	IRF07	96.00	0	100.00
IRF12	25	0	100.00	0	100.00	0	100.00	0	100.00	IRF10	96.00
IRF15	25	0	100.00	0	100.00	IRF12	96.00	0	100.00	0	100.00
ORF07	25	0	100.00	0	100.00	0	100.00	0	100.00	ORF10	88.00
ORF10	25	0	100.00	0	100.00	0	100.00	ORF15	96.00	0	100.00
ORF12	25	0	100.00	0	100.00	ORF12	92.00	ORF10	96.00	0	100.00
ORF15	25	0	100.00	0	100.00	0	100.00	0	100.00	ORF12	96.00
CF05	25	0	100.00	0	100.00	0	100.00	CF10	96.00	0	100.00
CF10	25	0	100.00	0	100.00	0	100.00	0	100.00	CF05	96.00
Total	275	0	100.00	0	100.00	4	99.27	7	97.46	9	96.73

Note: MCR represent the misclassification result; Acc represent the Accuracy.

## Data Availability

The datasets used or analysed during the current study are available from the corresponding author on reasonable request.

## Supplementary Materials

The following are available online at http://www.phmsociety.org/competition/09, Figure: Test diagram of gear failure, Data set: generic industrial gearbox data.
